# The resurrection of *Neohattoria* Kamim. (Jubulaceae, Marchantiophyta): a six decade systematic conflict resolved through a molecular perspective

**DOI:** 10.3897/phytokeys.50.4940

**Published:** 2015-06-16

**Authors:** Juan Larraín, Benjamin Carter, Blanka Shaw, Jörn Hentschel, Lynika S. Strozier, Tatsuwo Furuki, Jochen Heinrichs, Barbara Crandall-Stotler, John Engel, Matt von Konrat

**Affiliations:** 1 Science & Education, The Field Museum, 1400 South Lake Shore Drive, Chicago, IL 60605-2496, U.S.A.; 2 Department of Biology, Duke University, Durham, NC 27708-0338, U.S.A.; 3 Department of Systematic Botany with Herbarium Haussknecht and Botanical Garden, Friedrich Schiller University, Fürstengraben 1, 07743 Jena, Germany; 4 Natural History Museum & Institute, 955-2 Aoba-cho, Chuo-ku, Chiba-shi, Chiba 260-8682, Japan; 5 Ludwig-Maximilians-Universität München, Department für Biologie I, Systematische Botanik und Mykologie, GeoBio-Center, Menzinger Straße 67, 80638 München, Germany; 6 Southern Illinois University, Department of Plant Biology, Mail Code 6509, wCarbondale IL 62901-6509, U.S.A.

**Keywords:** DNA sequence data, *Frullania*, Frullaniaceae, Japan, *Jubula*, Jubulaceae, Lejeuneaceae, liverwort, *Nipponolejeunea*

## Abstract

The systematic placement of *Frullania herzogii* has been contentious since its description six decades ago. Over the years it has been interpreted as either a member of the genus *Frullania* or segregated into its own genus, *Neohattoria*, due to morphological similarities with both *Frullania* and *Jubula*. Here we provide molecular evidence that supports the recognition of the genus *Neohattoria* and its inclusion within the Jubulaceae, together with *Jubula* and *Nipponolejeunea*. Jubulaceae are placed sister to Lejeuneaceae rather than to the monogeneric Frullaniaceae.

## Introduction

The liverwort *Frullania herzogii* S.Hatt. was originally described by Hattori (1955) from a poor, sterile specimen collected on Mt. Hayachine in Iwate Prefecture, northern Honshu, Japan. Since that time the generic and even familial placement of the species has remained controversial. The species also has remained poorly known partially because of its seemingly limited distribution in the subalpine coniferous forest zones of Honshu and Hokkaido, Japan, and the Kuril Islands (Inoue et al. 1981, Stotler and Crandall-Stotler 1987). Hattori (1955) remarked that the leaf morphology, with acute teeth along the margin, differed from all the other Japanese *Frullania* Raddi species known by him. A few years later, in his monograph of Japanese Frullaniaceae, Kamimura (1961) erected the new genus *Hattoria* Kamim. to separate this taxon from other *Frullania* species. He stated that although his new genus superficially resembled species of *Cololejeunea* (Spruce) Schiffn. or *Frullania*, there was an important similarity between the branching patterns of *Hattoria herzogii* (S.Hatt) Kamim. and species in the genus *Jubula* Dumort. In both *Jubula* and *Hattoria*, the branches replace the lobule of the leaf at the point of insertion, and the leaf lobes are attached to both the main stem and to the branch. Although Kamimura (1961) noted the similarity of cell shape between *Hattoria* and *Frullania*, he considered the combination of branching architecture and leaf denticulation sufficient to recognize *Hattoria* as a distinct genus. A year later he had to give a new name, *Neohattoria* Kamim., to his recently described genus (Kamimura 1962), because of the almost simultaneous although earlier description of *Hattoria* by Schuster for a liverwort in the Lophoziaceae (Schuster 1961).

Later Schuster (1963), in a key for the Southern Hemisphere genera of liverworts, expanded the circumscription of *Neohattoria* to include two more species, *Frullania microscopica* Pearson from New Caledonia, and *Frullania parhamii* (R.M.Schust.) R.M.Schust. ex von Konrat, L.Söderstr. & A.Hagborg from Fiji. He based his taxonomic decision on the morphology of the reduced leaves on branch bases, the subfloral innovations, and the sharply delimited bracts and bracteoles of *Frullania microscopica*, and on the toothed leaf lobes of this species. Schuster (1963) did not provide any argument for placement of the Fijian *Frullania parhamii* in *Neohattoria*, other than the hyaline margins of the leaves that can be seen in this species and in *Frullania microscopica* (as inferred from the key). However, his key is restricted to the Southern Hemisphere and did not include the type of the genus, which completely lacks a hyaline border in leaf lobes. Schuster (1970) later expanded this generic concept even further, including the Australasian *Frullania rostrata* (Hook.f. & Taylor) Hook.f. & Taylor ex Gottsche, Lindenb. & Nees (as *Neohattoria australis* R.M.Schust.) and *Frullania hodgsoniae* von Konrat, Braggins, Hentschel & Heinrichs (as *Neohattoria rostrata* R.M.Schust.), the SE Asian Frullania junghuhniana Gottsche var. tenella (Sande Lac.) Grolle & S.Hatt. [as *Neohattoria perversa* (Steph.) R.M.Schust.], the New Caledonian *Frullania chevalieri* (R.M.Schust.) R.M.Schust. and *Frullania neocaledonica* J.J.Engel (as *Neohattoria caledonica* R.M.Schust.). Of these, *Frullania hodgsoniae* is now considered a member of Frullania subg. Diastaloba Spruce sect. Inconditum von Konrat, Hentschel & Heinrichs (von Konrat et al. 2010), while the rest of the taxa are currently included in Frullania subg. Microfrullania (R.M.Schust.) R.M.Schust. The current taxonomic placement of these taxa is based on both morphological (Hattori and Mizutani 1982, Schuster 1992) and molecular evidence (Hentschel et al. 2009, von Konrat et al. 2012).

Asakawa et al. (1979) demonstrated, based on chemical compound differences, that Jubulaceae *sensu lato* should be divided into three families, i.e. Jubulaceae, Frullaniaceae and Lejeuneaceae. This view has been confirmed by most molecular phylogenies published to date (e.g., Forrest et al. 2006, Heinrichs et al. 2005, 2007). Asakawa et al. (1979) listed 11 morphological characters that support the separation of Frullaniaceae and Jubulaceae, and placed *Neohattoria* together with *Jubula* in the Jubulaceae. Hattori (1982, 1984, 1986) and Hattori and Mizutani (1982) also accepted the separation between Jubulaceae and Frullaniaceae and argued that *Amphijubula* R.M.Schust., a genus formerly considered by Schuster (1970, 1980) as intermediate between *Jubula* and *Frullania*, should be placed within *Frullania*. This view was first held by Engel (1978), who had earlier reduced *Amphijubula* to a synonym of *Frullania*.

In 1987, Stotler and Crandall-Stotler published a thorough treatise of the taxonomic history of *Neohattoria herzogii* (S.Hatt.) Kamim. in the context of a detailed re-evaluation of its morphology, including the discovery of immature female inflorescences. In that contribution they came to the conclusion that this taxon should be considered within the circumscription of *Frullania*, although in its own subgenus, Frullania subg. Dentatilobi Stotler & Crand.-Stot. Their conclusion was based on both vegetative and reproductive characters, including the morphology of the bracts surrounding the female gametangia, lobule anatomy, leaf cell pattern, and the morphology of regenerants. Although they recognized that leaf-lobe insertion, branch morphology, and morphology of stylus are more similar to *Jubula* than to *Frullania*, they concluded that on the basis of the *Frullania*-like inflorescences and regenerants, *Neohattoria* should be synonomized with *Frullania*. This synonomy was adopted by Grolle and Meister (2004) who described a morphologically similar plant from Oligocene amber from Bitterfeld (Germany) as Frullania (subg. Dentatilobi) hamatosetacea Grolle. However, this fossil species appears morphologically closer to Frullania subg. Microfrullania than to *Neohattoria*, and this issue will be explored in detail in a forthcoming monograph of the latter subgenus.

Lack of useable specimens has previously precluded inclusion of *Neohattoria* in molecular phylogenetic studies. As a result of recent collecting activities, fresh material became available that allowed for successful DNA extraction and amplification. In the present study, we use molecular sequence data to investigate the phylogenetic position of *Neohattoria*. We investigate whether the genus should be placed in the Frullaniaceae or the Jubulaceae and evaluate whether molecular evidence supports the recognition of *Neohattoria* as a distinct genus.

## Methods

### Microscopy

For the production of microscopic images an Olympus BX51 microscope was used, equipped with both a QICAM Fast1394 camera from QIMAGING (Surrey, Canada), and a slide scanner (moving platform stage attached between the objectives and the condenser) from Objective Imaging Ltd. (Cambridge, UK). The software “Surveyor” from the latter company was used for the digitally rendered images.

### DNA extraction, PCR amplification and sequencing

We worked with two independent datasets to address two different questions, (1) what is the position of *Neohattoria* relative to the Frullaniaceae, Jubulaceae and Lejeuneaceae, and once we obtained results from these analyses, we asked (2) what is the position of *Neohattoria* within the Jubulaceae. For dataset 1 sequences were generated for two mitochondrial (*nad*1, *rps*3), and two chloroplast loci (*psb*A, *rbc*L), following DNA extraction, amplification and sequencing methods described by Shaw et al. (2003), and using primer sequences provided in Cooper et al. (2011). For dataset 2 we used the aforementioned plastid regions (*psb*A and *rbc*L) together with the nuclear ITS region following the methods described by Shaw et al. (2003), and the chloroplast *trn*L-*trn*F region, amplified and sequenced as described in von Konrat et al. (2012). All sequences were edited and manually aligned in PhyDE v0.9971 (www.phyde.de) following the alignment rules and hotspot definitions presented in Kelchner (2000), Olsson et al. (2009), and Borsch and Quandt (2009).

### Taxon sampling and outgroup selection

For dataset 1 seven species of *Radula* were selected as outgroup taxa following the results already published in recent liverwort phylogenies (Davis 2004, Forrest et al. 2006, Feldberg et al. 2014, Heinrichs et al. 2005, 2007). The same criteria were undertaken for dataset 2, including all taxa with sequences available in GenBank for *Jubula* and *Nipponolejeunea* S.Hatt. (Ahonen 2006, Ahonen et al. 2003, Konstantinova and Vilnet 2011, Pätsch et al. 2010, Wilson et al. 2004, 2007), using selected taxa of the Lejeuneaceae and species of *Frullania* as outgroup based on results from dataset 1. GenBank accession numbers for both newly generated sequences and for already published sequences are provided in Appendices 1 and 2 for datasets 1 and 2 respectively.

### Phylogenetic inferences

Both datasets were analysed with PartitionFinder v1.1.0 (Lanfear et al. 2012, 2014) to develop best-fit partitioning schemes and models of molecular evolution. Dataset 1 was partitioned setting one separate data block for each of the four genes used, each of them divided in three according to each codon position; introns and/or spacers were coded as extra partitions. Dataset 2 was partitioned in four parts, corresponding to the regions included only, without inner codon partition for the coding regions analysed. For dataset 1, phylogenetic reconstructions under maximum likelihood (ML) were performed in GARLI v2.01 (Zwickl 2006), setting up seven different models for the eleven partitions determined by PartitionFinder. Two independent searches each with 100 bootstrap replicates were made, and the 50% majority-rule consensus tree from all obtained trees was obtained with SumTrees v3.3.1 included in the package DendroPy v3.12.2 (Sukumaran and Holder 2010). Bayesian Posterior Probabilities analyses (PP) were executed in MrBayes v3.2.2 (Huelsenbeck and Ronquist 2001, Ronquist and Huelsenbeck 2003) also with the partitioned data set as given by PartitionFinder, and setting a different model for the individual partitions from the available options in MrBayes, with all characters given equal weight and gaps treated as missing data. The default settings of the program for a priori probabilities were used. Four runs, each with four MCMC chains (one million generations each) were run simultaneously, with the temperature of the heated chain set to 0.2 (default setting). Chains were sampled every 100 generations. Calculation of the consensus tree and posterior probabilities of clades was based on the set of trees sampled after the chains had converged, as observed graphically using Tracer v1.5 (Rambaut and Drummond 2007). For dataset 2, phylogenetic reconstructions under ML were performed in GARLI v2.01 and Bayesian analyses were executed with MrBayes v3.2.2 following the protocols as described above. For this dataset only three different partitions were suggested by PartitionFinder, and the models given by this software for each partition were incorporated into the settings of both the ML and the Bayesian analysis. Trees were edited and support values added using TreeGraph v2.0.54-364 beta (Stöver and Müller 2010).

## Results

**Figure 1.**
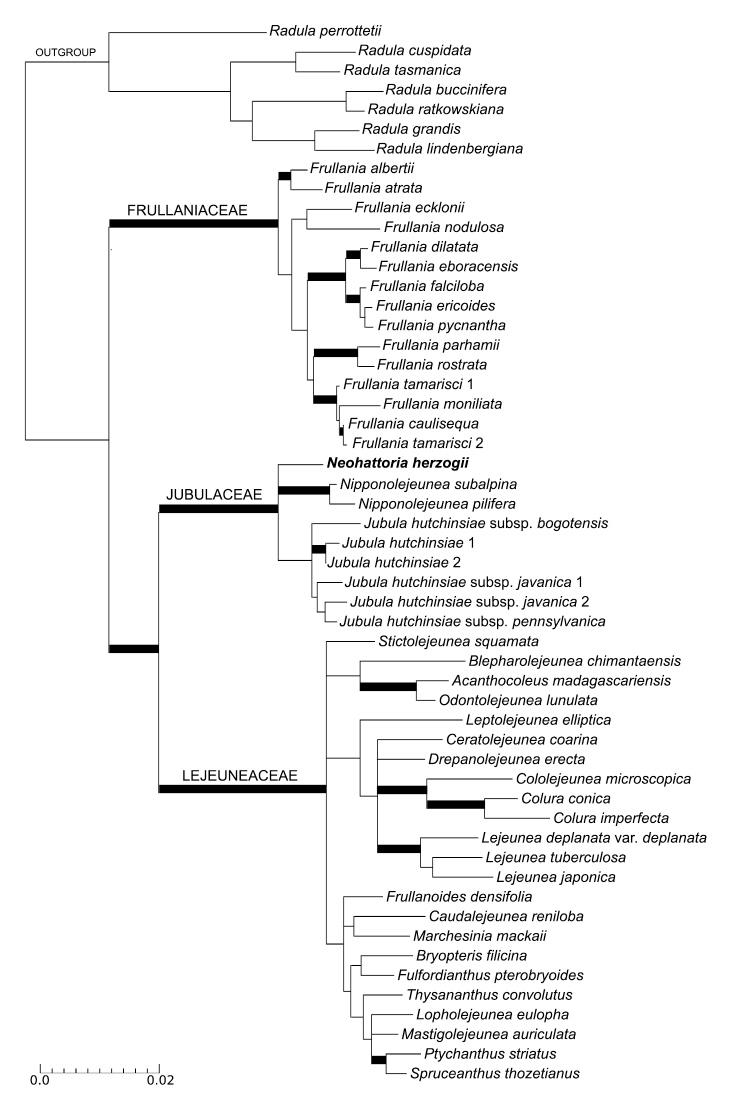
Maximum likelihood (ML) tree showing the systematic position of *Neohattoria* relative to the Jubulaceae, Frullaniaceae and Lejeuneaceae. Wide black branches indicate ML bootstrap support > 90 % and PP > 0.95.

The complete alignment for dataset 1 including all four regions mentioned above, with flanking areas pruned to avoid ambiguous readings, comprised 4818 characters for 54 accessions, of which 694 were parsimony informative. A total of 101 new sequences were generated for this study (Appendix 1). In the analysis of the *Neohattoria* sequences with accessions of the Frullaniaceae, Jubulaceae and Lejeuneaceae (dataset 1), *Neohattoria* is strongly supported (as defined by Pedersen et al. 2007) as one of three clades belonging to the Jubulaceae in both ML and Bayesian analyses, with accessions of *Nipponolejeunea*, resolved in a second clade and those of *Jubula*, in a third clade (Fig. 1), although the latter with low support (ML = 52, PP = 0.6). The Jubulaceae is resolved as sister to the Lejeuneaceae with strong support in both types of analysis. The position of the Frullaniaceae as sister to this latter clade (Jubulaceae + Lejeuneaceae) was strongly supported by the Bayesian analyses (PP = 1.0), but it was not recovered by the ML analyses. The Bayesian analyses also resolved *Neohattoria* as sister to the rest of the Jubulaceae (*Nipponolejeunea* + *Jubula*) with strong support (PP = 1.0).

**Figure 2.**
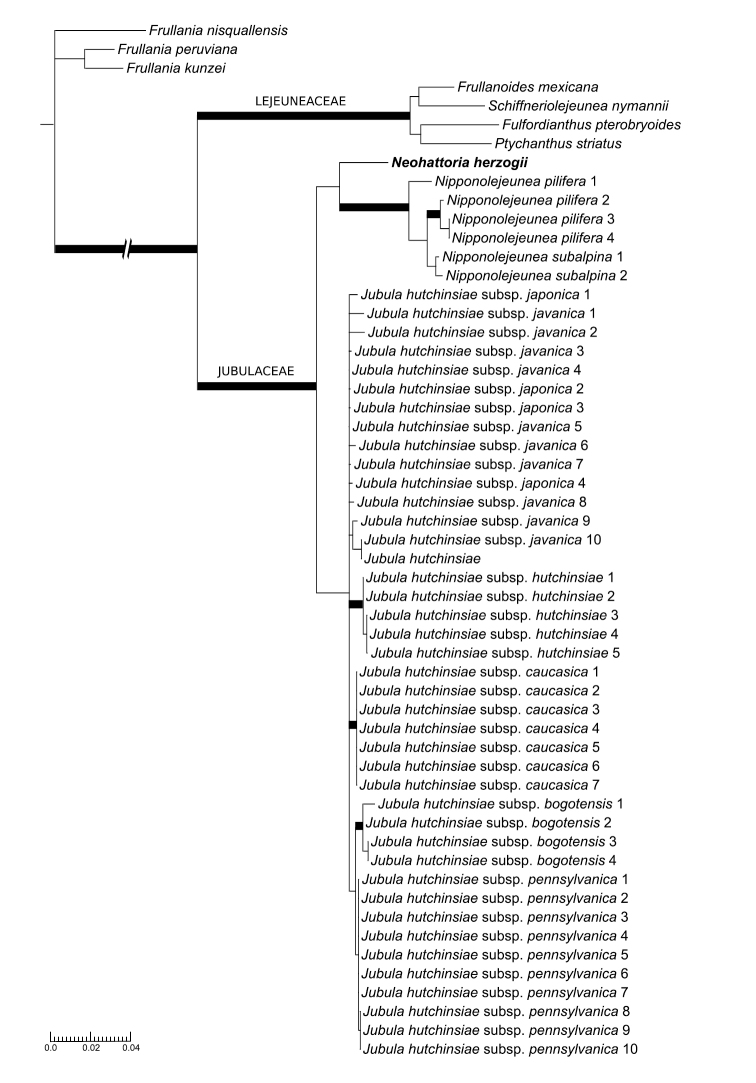
Maximum likelihood (ML) tree showing the systematic position of *Neohattoria herzogii* within the Jubulaceae. Only 1/2 of the length of the branch between the Frullaniaceae and the Lejeuneaceae/Jubulaceae clade is depicted. Wide black branches indicate ML bootstrap support > 90 % and PP > 0.95.

The complete alignment for dataset 2 including all four regions included, and after pruning the flanking areas to avoid ambiguous readings and deleting unalignable areas of the ITS region, comprised 3737 characters for 55 accessions, of which 548 were parsimony informative. The four different regions were not equally represented in the matrix, as shown in Appendix 2. The results of the analyses (Fig. 2) confirm with strong support the placement of *Neohattoria* within the Jubulaceae (ML = 100, PP = 1.0), and forming a sister clade to *Nipponolejeunea*, although recovered with strong support only by the Bayesian analysis (ML = 64, PP = 0.97). *Jubula* was resolved as the sister clade to the *Neohattoria*-*Nipponolejeunea* clade, although with low support (ML = 65, PP = 0.5).

**Figure 3.**
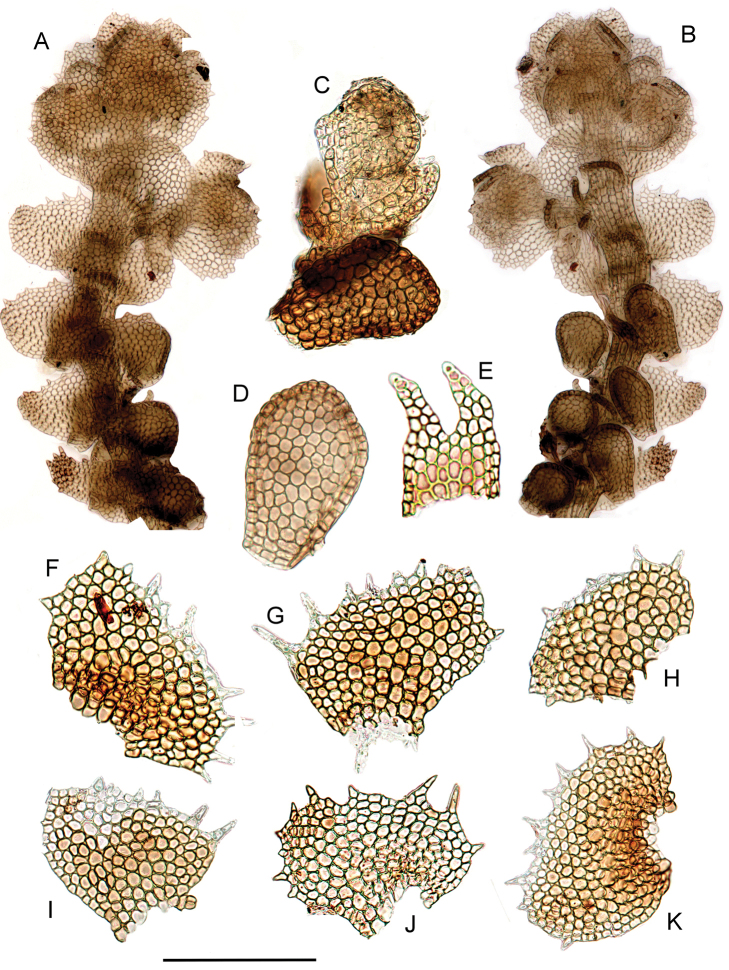
*Neohattoria herzogii*. **A** Habit, dorsal view **B** Habit, ventral view with distal lobules detached **C** Regenerant shoot originating from a detached lobule **D** Lobule **E** Underleaf **F–K** Leaves. All from Furuki 22673 (F). Scale bar: 350 µm (**A, B**), 200 µm (**C**), 180 µm (**D**), 300 µm (**E**), 150 µm (**F–K**).

The voucher of *Neohattoria herzogii* used for DNA extraction is illustrated in Figure 3.

## Discussion

Our molecular analyses support recognition of the genus *Neohattoria* as distinct from the genus *Frullania*, as first proposed by Kamimura (1961) almost 55 years ago. Moreover, our molecular analysis strongly supports its inclusion within the Jubulaceae, together with *Jubula* and *Nipponolejeunea*. A close relationship with *Jubula*, based on similarities in branch morphologies, was first suggested by Kamimura (1961, p. 94), and also accepted by Hattori et al. (1972). Inoue et al. (1981) provided new karyological, chemical and ecological data on *Neohattoria herzogii* and concluded that the biosystematic evidence collected suggested distance between *Jubula* and *Neohattoria*, but, nonetheless, retained *Neohattoria* in the Jubulaceae. While morphologically closer to *Jubula* than *Nipponolejeunea* to which it is sister, it is clearly not nested in the *Jubula* clade. This combination of molecular and morphological evidence, in fact, supports its recognition as a distinct genus in the Jubulaceae.

### Circumscription and relationships of the Jubulaceae

Our results strongly support the position of the Jubulaceae (containing *Jubula*, *Nipponolejeunea* and *Neohattoria*) sister to the Lejeuneaceae, and the Frullaniaceae as sister of the latter clade, although without significant support (Fig. 1). These results agree with several molecular phylogenies (e.g. Ahonen 2004, Forrest et al. 2006, Heinrichs et al. 2005, 2007). Thus the traditional view of a widely circumscribed Jubulaceae including *Frullania* is further rejected in this study.

These three families (Frullaniaceae, Jubulaceae and Lejeuneaceae) share several morphological characters, including the leaves divided into two (or three) parts [lobe, lobule (and stylus)], the beaked perianths, the sporophyte enclosed in a stalked true calyptra, the bistratose capsule wall, and the vertically aligned elaters that are attached to the valve apices (Crandall-Stotler et al. 2009, Gradstein et al. 2001, Schuster 1992). However, these characters need to be carefully evaluated to understand their evolution and their role in demonstrating the history of these lineages. In the past, *Jubula*, *Frullania* and members of the Lejeuneaceae were placed in a single taxonomic group (the subtribe Jubuleae), based largely on the similarities among their sporophytes (e.g. Müller 1915). Verdoorn (1930) argued that based on most characters (e.g., number of archegonia, seta form, and lobule ontogeny) *Jubula* belongs nearest to *Frullania*, which later lead Schuster (1992, p. 6) to describe *Jubula* as a “*bona-fide* genus of Jubulaceae [= Frullaniaceae]”. Mizutani (1961) was the first to propose that, except for the lobule structure, *Jubula* had no alignment with *Frullania*, and subsequently placed *Jubula* into the Lejeuneaceae. However, Asakawa et al. (1979) concluded that chemically, both *Jubula* and *Frullania* are quite different from *Lejeunea* Lib. species. Interestingly, the phylogenetic analysis by Crandall-Stotler and Stotler (2000) of 40 gametophyte and 21 sporophyte characters distributed among 34 liverwort families, resolved *Frullania asagrayana* Mont. as sister to a clade containing Jubula hutchinsiae (Hook.) Dumort. subsp. pennsylvanica (Steph.) Verd. and *Lejeunea cavifolia* (Ehrh.) Lindb. However, in the systematic treatment of the same work (Crandall-Stotler and Stotler 2000) Jubulaceae is presented as including both *Jubula* and *Frullania*, whereas the Lejeuneaceae is presented as a separate family, following accepted classifications of the time. The revised version of that classification, incorporating some recent molecular data, presents the Frullaniaceae, Jubulaceae and Lejeuneaceae as three separate families within the suborder Jubulineae (Crandall-Stotler et al. 2008, 2009), which is accepted here but with the transfer of *Neohattoria* from the Frullaniaceae to the Jubulaceae.

Assessing the importance of different morphological characters in circumscribing Frullaniaceae, Jubulaceae and Lejeuneaceae has been a difficult problem, but there are several characters that are consistent with the molecular phylogenetic results presented here. In most Lejeuneaceae a true stylus does not develop, but instead a single, unstalked slime papilla is formed at the junction of the lobule base and the stem, while in *Jubula* and *Neohattoria* there is a one- or two-celled filament terminated by a slime papilla in this position (Crandall-Stotler and Guerke 1980, Stotler and Crandall-Stotler 1987). Both types of structures are clearly different from those of the Frullaniaceae, where the stylus is always formed by more than two cells and is usually very conspicuous. The Jubulaceae and Frullaniaceae can be clearly differentiated from the Lejeuneaceae by the lobule, which is almost free from the larger dorsal lobe, and typically modified into an inflated, balloon-like to helmet-shaped sac whose aperture is directed either toward the shoot base or toward the stem, with the exception of *Nipponolejeunea* which has Lejeuneaceae-like lobules. Guerke (1978) hypothesised that *Jubula* was more advanced than *Frullania* on the basis that *Jubula* has many specialized characteristics e.g., a highly reduced stylus, seta, and foot, and features associated with the sporeling. In contrast, Schuster (1992, p. 9) stated that taxa such as *Amphijubula microcaulis* (Gola) R.M.Schust. (≡ *Frullania microcaulis* Gola), with a 16 + 4 seriate seta and monogynous gynoecia, diminish the distinctions between the two groups such that he prefers not to attempt a “subfamilial separation” at all. However, revision of the chemical, morphological, and ecological data provided support for the recognition of two subfamilies in the Jubulaceae (Guerke 1978, von Konrat 2004). Alternatively, Asakawa et al. (1979), on the basis of biochemical and morphological evidence, proposed two families: Jubulaceae (*Jubula*, *Neohattoria*) and Frullaniaceae (*Frullania*, *Steerea* S.Hatt., *Amphijubula*, and *Schusterella* S.Hatt.). Hattori (1982, 1984, 1986) and Hattori and Mizutani (1982) also accepted two families. This approach has been adopted in most recent hepatic floras and classifications (Paton 1999, Damsholt 2002, Casas et al. 2009, Crandall-Stotler et al. 2009, Frey and Stech 2009).

Schuster (1980, 1992) questioned the division into two families and argued that only the single family Jubulaceae should be recognized, but commented that this area of classification remains replete with ambiguities and contradictions. Interestingly, he also suggested that there was a possibility that *Neohattoria* might share a closer affinity to Jubulopsidaceae (= Lepidolaenaceae) than to Jubulaceae (Schuster 1996), a view first expressed when Grolle (1966) transferred *Jubula novae-zelandiae* E.A.Hodgs. & S.W.Arnell, which is the generitype of *Jubulopsis* R.M.Schust., to *Neohattoria*. However, recent molecular analyses (e.g., Heinrichs et al. 2005, Forrest et al. 2006) have demonstrated that *Jubulopsis* (= *Lepidolaena*) is far removed from the Jubulaceae.

Morphologically, the monogeneric Frullaniaceae can be differentiated from the Jubulaceae by: (1) plants usually with conspicuous secondary pigmentation, often reddish; (2) initial leaves of branches either trifid or bifid; and (3) spores with rosette-like protrusions. Conversely, in the Jubulaceae the plants are: (1) soft and without secondary pigmentation (thus usually dull green to pale brown); (2) the initial leaves of branches are small, subtriangular, and never tri- or bifid; and (3) the spores without rosette-like protrusions. The first two of these characters support the placement of *Neohattoria* within Jubulaceae rather than Frullaniaceae (spores remain unknown in *Neohattoria*).

Chemically, *Frullania* species in general, produce significant amounts of sesquiterpene lactones, diterpenoids, and bibenzyl derivatives, which are considered important chemosystematic markers of the group (Asakawa et al. 1981, 1983, 1987, Kraut et al. 1994). On the other hand, cyclocolorenone and maalioxide have been isolated as major components of Jubula hutchinsiae (Hook.) Dumort. subsp. japonica (Steph.) Horik. & Ando (Asakawa et al. 1979); interestingly cyclocolorenone is also widely distributed in the Porellaceae. In contrast, no members of *Jubula* or *Frullania* produce paraffinic hydrocarbons which are characteristic for *Neohattoria* (Inoue et al. 1981).

Interestingly, Schuster (1996) suggested that there was a possibility that *Neohattoria* might share a closer affinity to Jubulopsidaceae (= Lepidolaenaceae) than to Jubulaceae. This view was first expressed when Grolle (1966) transferred *Jubula novae-zelandiae* E.A.Hodgs. & S.W.Arnell, which is the type species of *Jubulopsis* R.M.Schust., to *Neohattoria*. However, preliminary unrooted trees made for this contribution including *Ascidiota* C.Massal., *Gackstroemia* Trevis., *Goebeliella* Steph., *Lepidogyna* R.M.Schust., *Lepidolaena* Dumort. (= *Jubulopsis*) and *Porella* L. together with representatives outside the Porellales, showed *Neohattoria* far away from Lepidolaenaceae but within Jubulaceae (results not depicted). These results are basically the same as the ones observed in recent molecular phylogenies (e.g. Heinrichs et al. 2005, Forrest et al. 2006), demonstrating that these groups are only distantly related to either the Jubulaceae or the Frullaniaceae.

### Circumscription and relationships of *Neohattoria*

Our results place *Neohattoria* in the Jubulaceae with strong support, together with *Nipponolejeunea* and *Jubula*. Within the Jubulaceae, *Neohattoria* is resolved as sister to *Nipponolejeunea*, and this latter clade sister to *Jubula*, although this relationship is sensitive to taxon sampling (cf. Figs 1 and 2), and not strongly supported in the analyses. When describing the genus *Hattoria* (later renamed *Neohattoria*), Kamimura (1961) conceived it as a monotypic genus containing only the Japanese endemic *Neohattoria herzogii*. The singularity of this taxon was well described and illustrated, highlighting its closer affinities to *Jubula* instead of *Frullania*, mostly because of its branching pattern and leaf insertion: “[…] *the branch replaces the lobule of leaf in origin and the lobe is inserted partly to the stem and partly to the branch. The first leaf and underleaf of branches are much deformed, being the “Vorblätter” of Verdoorn (1930)*.” (Kamimura 1961, p. 94). The characteristic combination of traits that led Kamimura to describe this new genus vanished when Schuster (1963, 1970) added more species in the circumscription of *Neohattoria* as explained above. Schuster (1970) still recognized the taxonomic singularity of *Neohattoria herzogii* when placing it in its own subgenus within *Neohattoria*, but failed to see the relationships of this taxon with other *Jubula* species, precisely because of his wide concept of *Neohattoria* that includes members of Frullania subg. Microfrullania and Frullania subg. Diastaloba.

Oil-bodies in *Neohattoria* are homogenous, usually more than ten per cell, and similar in size to chloroplasts (Hattori et al. 1972, Inoue et al. 1981). Hattori et al. (1972) reported 10–20 oil-bodies per leaf lobe median cell for *Neohattoria herzogii* and later Inoue et al. (1981, p. 25) reported a similar number “usually 7–15 per leaf-lobe cell (rarely up to 22)”. Hattori et al. (1972) stated that oil-bodies of *Neohattoria* are hyaline and homogenous, and Inoue et al. (1981) recorded in their specimen of *Neohattoria* that the oil-bodies were completely colourless and homogenous. However, they noted that sometimes they were faintly papillose with a few distinct granules; Inoue et al. (1981) were uncertain if this was due to degeneration of the oil-bodies. Reports of oil-body numbers for *Jubula* are ambiguous: although Guerke (1979) and Paton (1999) suggested they range between 3–7 in all *Jubula* taxa, Schuster (1992) stated that the oil-bodies are numerous in the North American material of *Jubula pennsylvanica* (≡ Jubula hutchinsiae subsp. pennsylvanica), ranging from 6–16 per cell, and Mizutani (1961) reported 2–10 for Japanese *Jubula*. All authors agree that the oil-bodies in *Jubula* are faintly granular or homogeneous. In *Nipponolejeunea*, on the other hand, the oil-bodies range between 3–5(7) per cell, are hyaline to somewhat grayish, and are formed by 15–20 internal oil-globules (Mizutani 1961). In *Frullania* the oil-bodies are usually larger, finely to coarsely papillose rather than smooth, and few per cell, with their number generally increasing from the leaf-lobe marginal cells to the basal cells, except in the species that have basal ocelli; however, this number rarely reaches the number of oil-bodies seen in *Neohattoria* or *Jubula*. The average number of oil-bodies from the 22 species studied by von Konrat (2004) is 4.3 per median lobe cell. One remarkable exception is the North American species *Frullania stylifera* (R.M.Schust.) R.M.Schust., which has up to 16 oil-bodies per median cell (von Konrat 2004). A survey of over sixty species (including literature data) suggests that this is a rare condition in the genus (von Konrat 2004). Schuster (1992) described the oil-bodies of *Frullania* as formed of numerous oil-globules and usually appearing coarsely to finely papillose, the only exception being the oil-bodies of Frullania subg. Microfrullania, which are smooth and frequently appear as almost homogeneous oil-droplets (von Konrat 2004). The oil-bodies of *Neohattoria* then appear closer to the other Jubulaceae genera in appearence (although smooth, homogeneous oil-bodies are also seen in Frullania subg. Microfrullania) and number, notwithstanding the number reported for *Nipponolejeunea* and some reports of *Jubula* taxa with fewer oil-body numbers.

### Nomenclatural novelties

***Neohattoria*** Kamim., Journal of Japanese Botany 37: 218. 1962.

≡ Frullania subg. Dentatilobi Stotler & Crand.-Stotl., Memoirs of The New York Botanical Garden 45: 542. 1987 (“*Dentatiloba*”). **syn.nov.** – Type: *Frullania herzogii* S.Hatt.

## Appendix 1

Voucher information for data set 1. Information is presented in the following order: taxon name, collector followed by collection number (herbarium acronyms follow Holmgren et al. 1990), country: region (if known), GenBank accesion numbers (*psb*A/*rbc*L/*rps*3/*nad*1). Lacking sequences are indicated by a dash (—). New sequences generated for this study are marked by an asterisk (*).

*Acanthocoleus madagascariensis* (Steph.) Kruijt, Pócs 97145/AA (GOET), Uganda, EF011843/DQ983649/—/—; *Blepharolejeunea chimantaensis* van Slageren & Kruijt, Pócs & Rico 00234/A (F), Venezuela, KF851876/—/—/KF852465; *Bryopteris filicina* (Sw.) Nees, Churchill, Magombo & Price 19855 (NY), Bolivia, AY607930/DQ439681/KF851576/KF852481; *Caudalejeunea reniloba* (Gottsche) Steph., Pócs et al. 01090/AB (F), Australia, KF851845/KF852294/KF851541/KF852441; *Ceratolejeunea coarina* (Gottsche) Schiffn., Zartman 1235.1 (DUKE), Brazil, AY607934/AY608026/—/KF852489; *Cololejeunea microscopica* (Taylor) Schiffn., Long & Rothero 37789 (E), Scotland: Wester Ross, KF851954/KF852386/KF851651/KF852552; *Colura conica* (Sande Lac.) K.I.Goebel, Pócs & Streimann 9986/W (F), Australia: Queensland, KM817490*/KM817513*/KM817536*/KM817462*; *Colura imperfecta* Steph., Pócs & Pócs 07019/A (F), Thailand, KF851881/KF852327/—/KF852469; *Drepanolejeunea erecta* (Steph.) Mizut., Long 28691 (E), Bhutan, JF513393/JF513452/KF851515/JF513342; *Frullania albertii* Steph., Davis 295 (DUKE), Ecuador, AY607942/DQ439685/KM817549*/KM817477*; *Frullania atrata* (Sw.) Nees ex Mont., Dauphin 3306 (F), Costa Rica, KM817491*/—/KM817540*/KM817466*; *Frullania caulisequa* (Nees) Mont., Karst, Shaw & Gibbs 022 (DUKE), USA: North Carolina, KM817500*/KM817526*/KM817553*/KM817481*; *Frullania dilatata* (L.) Dumort., Stotler 4666 (SIU), Portugal, KM817502*/KM817528*/KM817555*/KM817482*; *Frullania eboracensis* Lehm., Stotler 80-4354 (ABSH), USA: Illinois, AY688827/AY688779/KM817547*/KM817475*; *Frullania ecklonii* (Spreng.) Spreng. ex Gottsche, Lindenb. & Nees, Pócs 02030/W (F), Kenya, KM817488*/KM817510*/KM817533*/KM817459*; *Frullania ericoides* (Nees) Mont., Long 35167 (E), China: Yunnan, KM817486*/KM817507*/KM817531*/KM817456*; *Frullania falciloba* Taylor ex Lehm., Engel, von Konrat & Braggins 26837 (F), New Zealand, KM817489*/KM817511*/KM817534*/KM817460*; *Frullania moniliata* (Reinw., Blume & Nees) Mont., Mizutani s.n. (ABSH), Japan, AY507484/AY507401/KM817548*/KM817476*; *Frullania nodulosa* (Reinw., Blume & Nees) Nees, Pócs & Pócs 03261/A (F) Fiji, KM817492*/KM817517*/KM817541*/KM817467*; *Frullania parhamii* (R.M.Schust.) R.M.Schust. ex von Konrat, L.Söderstr. & A.Hagborg, von Konrat, Braggins & Naikatini 6/16-5 (F), Fiji, —/KM817516*/KM817539*/KM817465*; *Frullania pycnantha* (Hook.f. & Taylor) Taylor ex Gottsche, Lindenb. & Nees, von Konrat 99/409 (F), New Zealand, KM817499*/KM817525*/—/KM817480*; *Frullania rostrata* (Hook.f. & Taylor) Hook.f. & Taylor ex Gottsche, Lindenb. & Nees, Engel, von Konrat & Braggins 27770 (F), New Zealand, —/KM817512*/KM817535*/KM817461*; *Frullania tamarisci* (L.) Dumort. 1, Stotler 4661 (SIU), Portugal: Sintra, KM817501*/KM817527*/KM817554*/—; *Frullania tamarisci* 2, Long 35371 (E), France, KM817487*/KM817508*/KM817532*/KM817457*; *Frullanoides densifolia* Raddi, Gradstein 10171 (GOET), Ecuador, KF851930/KF852371/KF851634/KF852530; *Fulfordianthus pterobryoides* (Spruce) Gradst., Gradstein & Varon 11069 (GOET), Colombia, KF851931/KF852372/KF851635/KF852531; *Jubula hutchinsiae* (Hook.) Dumort. 1, Long 29077 (E), UK: England, —/KM817509*/—/KM817458*; *Jubula hutchinsiae* 2, Drehwald 3007 (GOET), Portugal, EF011746/AY548101/—/—; Jubula hutchinsiae subsp. bogotensis (Steph.) Verd., Gradstein s.n. (GOET), Mexico, EF011758/AY548100/—/—; Jubula hutchinsiae subsp. javanica (Steph.) Verd. 1, Konstantinova & Savchenko K479/1-07 (F), Russia, —/KM817506*/KM817542*/KM817468*; Jubula hutchinsiae subsp. javanica 2, Kodama s.n. (ABSH), Japan: Wakayama Pref., AY507492/AY507408/KF851585/JF513366; Jubula hutchinsiae subsp. pennsylvanica (Steph.) Verd., Risk 11005 (DUKE), USA, AY607954/KM817523*/KM817550*/—; Lejeunea deplanata Nees var. deplanata, Shaw F533 (DUKE), USA: North Carolina, KM817498*/KM817524*/KM817552*/KM817479*; *Lejeunea japonica* Mitt., Bakalin s.n. (F), Russia, —/KM817518*/KM817543*/KM817469*; *Lejeunea tuberculosa* Steph., Long 28596 (E), Bhutan, JF513394/JF513453/KF851518/JF513344; *Leptolejeunea elliptica* (Lehm.) Besch., Yamaguchi s.n. (F), Japan, KM817485*/KM817515*/KM817538*/KM817464*; *Lopholejeunea eulopha* (Taylor) Schiffn., Pócs et al. 08036/U (F), Fiji, KF851868/KF852314/—/—; *Marchesinia mackaii* (Hook.) Gray, Buryova 2181 (DUKE), UK: Wales, —/KF852356/KF851619/KF852515; *Mastigolejeunea auriculata* (Wilson) Steph., Shaw 6222 (DUKE), USA: Alabama, KF851917/KF852359/KF851622/KF852518; *Neohattoria herzogii* (S.Hatt.) Kamim., Furuki 22673 (F), Japan: Honshu, KM817504*/KM817530*/KM817557*/KM817484*; *Nipponolejeunea pilifera* (Steph.) S.Hatt., Ohnishi 5975 (HIRO), Japan, AM396291/AM392293/—/—; *Nipponolejeunea subalpina* (Horik.) S.Hatt., Ohnishi 5611 (GOET), Japan, AM396290/AM392292/—/—; *Odontolejeunea lunulata* (F.Weber) Schiffn., Picon et al. 00227/CE (F), Venezuela, —/KM817514*/KM817537*/KM817463*; *Ptychanthus striatus* (Lehm.) Nees, Pócs & Pócs 03288/O (F), Fiji, KF851872/KF852318/KF851558/KF852460; *Radula buccinifera* (Hook.f. & Taylor) Taylor ex Gottsche, Lindenb. & Nees, Engel, von Konrat & Braggins 23569 (F), New Zealand, KM817495*/KM817521*/KM817545*/KM817472*; *Radula cuspidata* Steph., Engel & von Konrat 23517 (F), New Zealand, KM817496*/—/KM817546*/KM817473*; *Radula grandis* Steph., Engel, von Konrat & Braggins 24847 (F), New Zealand, KM817494*/KM817520*/KM817544*/KM817471*; *Radula lindenbergiana* Gottsche ex C.Hartm., Stotler 4656 (SIU), Portugal, KM817503*/KM817529*/KM817556*/KM817483*; *Radula perrottetii* Gottsche ex Steph., Mizutani 15030 (F), Japan, —/DQ439700/KM817551*/KM817478*; *Radula ratkowskiana* K.Yamada, Engel, von Konrat & Braggins 24365 (F), New Zealand, KM817497*/KM817522*/—/KM817474*; *Radula tasmanica* Steph., Engel, von Konrat & Braggins 24874 (F), New Zealand, KM817493*/KM817519*/—/KM817470*; *Spruceanthus thozetianus* (Gottsche & F.Muell.) B.M.Thiers & Gradst., Pócs 01107/M (GOET), Australia, AM396273/AM384877/—/—; *Stictolejeunea squamata* (Willd. ex F.Weber) Schiffn., Dauphin & Gonzalez 2134 (GOET), Costa Rica: Alajeula, KF851951/—/—/KF852549; *Thysananthus convolutus* Lindenb., Gradstein 10205 (GOET), Indonesia: Java, KF851953/DQ983737/KF851650/KF852551.

## Appendix 2

Voucher information for data set 2. Information is presented in the following order: taxon name, collector followed by collection number (herbarium acronyms follow Holmgren et al. 1990), country: region (if known), GenBank accesion numbers (ITS region/rbcL/trnL-F/psbA). Lacking sequences are indicated by a dash (—). New sequences generated for this study are marked by an asterisk (*).

*Frullania kunzei* (Lehm.) Lehm. & Lindenb., Costa & Gradstein 3769 (GOET), Brazil, FJ380536/FJ380863/FJ380387/FJ380697; *Frullania nisquallensis* Sull., Doyle 11001 (GOET), USA, FJ380503/FJ380826/FJ380349/FJ380661; *Frullania peruviana* Gottsche, Schaefer-Verwimp & al. 24356 (GOET), Ecuador, FJ380543/FJ380870/FJ380394/FJ380704; *Frullanoides mexicana* van Slageren, Burghardt 4421a, Mexico, DQ987366/DQ983682/DQ987464/EF011851; *Fulfordianthus pterobryoides* (Spruce) Gradst., Dauphin 2518, Costa Rica, AM237145/DQ983684/AM237198/EF011832; *Jubula hutchinsiae* (Hook.) Dumort., Ahonen, Huttunen et Virtanen 3190 (H), Taiwan, AY125350/AY125946/AY144477/—; Jubula hutchinsiae subsp. bogotensis (Steph.) Verd. 1, Gradstein s.n. (GOET), Mexico: Veracruz, FN396818/—/FN398013/—; Jubula hutchinsiae subsp. bogotensis 2, Gradstein s.n. (GOET?), Mexico, DQ987273/AY548100/DQ987388/AM396281; Jubula hutchinsiae subsp. bogotensis 3, Gradstein 9449 (GOET), Costa Rica, FN396817/—/FN398012/—; Jubula hutchinsiae subsp. bogotensis 4, Frahm et al. 1313 (GOET), Peru, FN396816/—/—/—; Jubula hutchinsiae subsp. caucasica Konstant. & Vilnet 1, Konstantinova K456-5-07 (KPABG), Russia: Caucasus, JN836964/—/JN836974/—; Jubula hutchinsiae subsp. caucasica 2, Konstantinova K429-3-08 (KPABG), Russia: Caucasus, JN836961/—/JN836971/—; Jubula hutchinsiae subsp. caucasica 3, Konstantinova K462-1-08 (KPABG), Russia: Caucasus, JN836960/—/JN836970/—; Jubula hutchinsiae subsp. caucasica 4, Konstantinova K463-1-07 (KPABG), Russia: Caucasus, JN836962/—/JN836972/—; Jubula hutchinsiae subsp. caucasica 5, Konstantinova K371-1-08 (KPABG), Russia: Caucasus, JN836958/—/JN836968/—; Jubula hutchinsiae subsp. caucasica 6, Konstantinova K446-7-08 (KPABG), Russia: Caucasus, JN836959/—/JN836969/—; Jubula hutchinsiae subsp. caucasica 7, Konstantinova K443-14-08 (KPABG), Russia: Caucasus, JN836963/—/JN836973/—; Jubula hutchinsiae subsp. hutchinsiae 1, Long 29077 (GOET), UK: Devon, FN396813/—/FN398010/—; Jubula hutchinsiae subsp. hutchinsiae 2, Long 35296 (GOET), UK: Wales, FN396814/—/FN398011/—; Jubula hutchinsiae subsp. hutchinsiae 3, Schaefer-Verwimp & Verwimp 25675 (GOET), Portugal: Madeira, FN396811/—/FN397099/—; Jubula hutchinsiae subsp. hutchinsiae 4, Schaefer-Verwimp & Verwimp 25796 (GOET), Portugal: Boaventura, FN396812/—/FN398009/—; Jubula hutchinsiae subsp. hutchinsiae 5, Drehwald & Reiner-Drehwald 3007 (GOET), Portugal, DQ987260/AY548101/DQ987380/AM396282; Jubula hutchinsiae subsp. japonica (Steph.) Horik. & Ando 1, Koponen et al. 54308 (H), China, AY125342/AY125938/AY144479/—; Jubula hutchinsiae subsp. japonica 2, Inoue BSE755 (GOET), Japan: Kochi, FN396809/—/—/—; Jubula hutchinsiae subsp. japonica 3, Gradstein & Mizutani 2958 (GOET), Japan: Miyazaki, FN396810/—/FN397098/—; Jubula hutchinsiae subsp. japonica 4, Bakalin P-68-10-08 (KPABG), Russia: Primorsky Kray, JN836967/—/JN836977/—; Jubula hutchinsiae subsp. javanica (Steph.) Verd. 1, Zhu et al. 3361 (HSNU), China: Hainan, FN396800/—/—/—; Jubula hutchinsiae subsp. javanica 2, Zhu et al. 20050903-7a (HSNU), China: Hainan, FN396801/—/—/—; Jubula hutchinsiae subsp. javanica 3, Long 34765 (GOET), China: Yunnan, FN396805/—/FN397095/—; Jubula hutchinsiae subsp. javanica 4, Pocs 98105/C (GOET), Viet Nam: Vin-Phuc, FN396807/—/—/—; Jubula hutchinsiae subsp. javanica 5, Pocs & Tran Ninh 98103/A2 (GOET), Viet Nam: Vin-Phuc, FN396808/—/FN397097/—; Jubula hutchinsiae subsp. javanica 6, Schaefer-Verwimp & Verwimp 18870/A (GOET), Malaysia: Pahang, FN396802/—/FN397094/—; Jubula hutchinsiae subsp. javanica 7, Zhu 555 (HSNU), China: Fujian, FN396806/—/FN397096/—; Jubula hutchinsiae subsp. javanica 8, Bakalin Kor-12-6-08 (KPABG), South Korea, JN836966/—/JN836976/—; Jubula hutchinsiae subsp. javanica 9, Schaefer-Verwimp & Verwimp 18935 (GOET), Malaysia: Pahang, FN396803/—/—/—; Jubula hutchinsiae subsp. javanica 10, Wang 685B (HSNU), China: Yunnan, FN396804/—/—/—; Jubula hutchinsiae subsp. pennsylvanica (Steph.) Verd. 1, Buck 39060 (H?), USA: West Virginia, AY776308/AY776303/AY776309/—; Jubula hutchinsiae subsp. pennsylvanica 2, Davison 5045 (UNAF), USA: Alabama, FN396819/—/—/—; Jubula hutchinsiae subsp. pennsylvanica 3, Davison 5201 (UNAF), USA: West Virginia, FN396821/—/FN398015/—; Jubula hutchinsiae subsp. pennsylvanica 4, Davison 4707 (UNAF), USA: Alabama, FN396822/—/FN398016/—; Jubula hutchinsiae subsp. pennsylvanica 5, Davison 3775a (UNAF), USA: Alabama, FN396823/—/FN398017/—; Jubula hutchinsiae subsp. pennsylvanica 6, Davison & Risk 2537 (UNAF), USA: Kentucky, FN396820/—/FN398014/—; Jubula hutchinsiae subsp. pennsylvanica 7, Konstantinova ACH-3-92 (KPABG), USA, JN836965/—/JN836975/—; Jubula hutchinsiae subsp. pennsylvanica 8, Davison 4690 (UNAF), USA, Alabama, FN396824/—/FN398018/—; Jubula hutchinsiae subsp. pennsylvanica 9, Hyatt 8212 (UNAF), USA: North Carolina, FN396825/—/FN398019/—; Jubula hutchinsiae subsp. pennsylvanica 10, Davison s.n. (UNAF), USA: North Carolina, FN396826/—/FN398020/—; *Neohattoria herzogii* (S.Hatt.) Kamim., Furuki 22673 (F), Japan: Honshu, KM817455*/KM817530*/KM817505*/KM817504*; *Nipponolejeunea pilifera* (Steph.) S.Hatt. 1, Ohnishi 5975 (HIRO), Japan, —/AM392293/FJ380228/AM396291; *Nipponolejeunea pilifera* 2, Higuchi 41359 (H?), Japan, AY776307/AY776304/AY776310/—; *Nipponolejeunea pilifera* 3, Masuzaki 510 (HIRO), Japan: Yakushima Is., —/AB476588/—/—; *Nipponolejeunea pilifera* 4, Ohnishi s.n. (H), Japan, AY125341/AY125937/AY144478/—; *Nipponolejeunea subalpina* (Horik.) S.Hatt. 1, Ohnishi 5611 (HIRO), Japan, DQ987289/AM392292/FJ380227/AM396290; *Nipponolejeunea subalpina* 2, Higuchi 41358 (H?), Japan, AY776306/AY776305/AY776311/—; *Ptychanthus striatus* (Lehm.) Nees, Gradstein 10217, Indonesia: Java, DQ987297/DQ983723/DQ987403/EF011777; *Schiffneriolejeunea nymannii* (Steph.) Gradst. & Terken, Gradstein et al. 10321, Malaysia, DQ987320/DQ983725/DQ987424/EF011801.
